# Die Bedeutung der präoperativen Immunhistochemie bei Patientinnen mit Endometriumkarzinomen – welche Parameter sind entscheidend?

**DOI:** 10.1007/s00066-021-01862-6

**Published:** 2021-11-11

**Authors:** S. Marnitz

**Affiliations:** Klinik für Radioonkologie, Cyberknife und Strahlentherapie, Medizinische Fakultät, Universität zu Köln, Kerpener Str. 62, 50937 Köln, Deutschland

## Hintergrund und Fragestellung

Die genomische Klassifikation des Endometriumkarzinoms [2] hat uns vor Augen geführt, dass die Zweiteilung in Typ I und Typ II sowie die klinischen Faktoren wie Myometriuminfiltration, Grading, Lymphangiose (L), Hämangiose (V), Tumorgröße und das Alter der Patientin die Biologie des Tumors nicht ausreichend beschreiben. Das Ergebnis sind insuffiziente Risikostratifizierungen („low risk, intermediate, high intermediate, high risk“), auf deren Basis Therapieentscheidungen für Tausende von Patientinnen getroffen wurden und z.T. auch weiterhin werden. Zahlreiche klinisch bedeutsame Fragestellungen zum Wert der adjuvanten Therapie, wie z. B. perkutane Therapie (EBRT) vs. Brachytherapie (BT); Radiochemotherapie (RCT) vs. Radiotherapie; RCT vs. Chemotherapie + BT etc. [3–12], führten alle zu inkonklusiven Ergebnissen, da die Patientenselektion auf der Basis der oben aufgeführten klassischen Risikoeinteilungen die Biologie der Erkrankung unvollständig beschreibt [13–15]. Statt der klinischen sind nämlich die genomischen Faktoren entscheidend. Die Einteilung in (1) POLE-Mutationen, (2) Tumoren ohne spezielles molekulares Profil („non specific molecular profile“ [NSMP]), (3) die mikrosatelliteninstabilen Tumoren (MSI = „mismatch repair deficiency“ [MMRd]) und (4) die p53-mutierten Tumoren hat eine neue, bisher unbekannte Welt eröffnet.

Folgerichtig machte sich die PORTEC-Gruppe an die Reanalyse ihrer Studienergebnisse, erhob von den in Studien behandelten Patientinnen dieses molekulare Profil und korrelierte es mit dem onkologischen Ergebnis [16]. Dabei zeigte sich, dass POLE-Mutationen zu einem sehr günstigen onkologischen Outcome passen, NSMP- und MSI-Patientinnen eine intermediäre Prognose aufweisen und p53-Patientinnen eine deutlich ungünstigere Gruppe darstellen. Es existieren Schnittmengen mit den klassischen klinischen Prognosefaktoren, aber auch mit vielen Patientinnen, die keiner adjuvanten Therapie bedurft hätten (POLE-Mutationen), und Patientinnen, die p53-Mutationen aufweisen und in der Vergangenheit eher das Risiko einer Untertherapie hatten.

Auf der Basis dieser Daten hat die europäische Leitliniengruppe bereits ihre Risikoeinteilung inklusive der Therapieempfehlungen angepasst [17]. Für die deutsche Leitlinie steht dies noch aus [18]. Da bisher keine Level-I- oder -II-Evidenz vorliegt, sind die logische Konsequenz nun Studien, die das genomische Profil der Patientin zur Entscheidungsgrundlage machen, wie z. B. die laufende PORTEC-4a-Studie (ClinicalTrials.gov Identifier: NCT03469674). Die nachfolgend zu kommentierende Arbeit diskutiert, inwieweit zusätzliche immunhistochemische (IHC) Parameter, wie das Adhäsionsmolekül L1CAM und das in die ESGO/ESTRO-Leitlinien aufgenommene p53 sowie „alte Bekannte“ wie der Hormonrezeptorstatus, zu einer weiteren Verfeinerung der Prognosegruppen und somit der Therapieempfehlungen beitragen können.

## Material und Methoden

Die Daten von** >** 760 Patientinnen aus 10 europäischen Kliniken wurden ausgewertet. Das mittlere Follow-up betrug 5,5 Jahre. 71 % der Patientinnen wurden präoperativ als G1/2 diagnostiziert, 89 % wiesen eine endometrioide Histologie auf.

## Ergebnisse

Die präoperative Immunhistochemie zeigte p53_abn_-Befunde bei 112 (14,7 %), L1CAM+ bei 79 (10,4 %) und einen negativen ER/PR-Status bei 151 (20 %) der Patientinnen. Bei 493 (65 %) Patientinnen war eine Lymphadenektomie erfolgt, 53 Patientinnen (11 %) wiesen Lymphknotenmetastasen auf. Die Indikation zur adjuvanten Therapie wurde bei 347 (46 %) gestellt. Von diesen 112-mal eine VBT, 104-mal eine EBRT und 93-mal eine VBT + EBRT. Eine Chemotherapie bzw. Radiochemotherapie erhielten 38 bzw. 26 Patientinnen. 102 (13,4 %) der 105 (13,8 %) Patientinnen, die ein Rezidiv entwickelten, starben an der Erkrankung. 12 % der hier untersuchten Patientinnen wiesen immunhistochemisch ein abnormes p53 auf; dies gilt als valider Surrogatparameter zur p53-Mutationsuntersuchung. p53_abn_ erwies sich als wichtigster Prognosefaktor.

**Figure 1:**
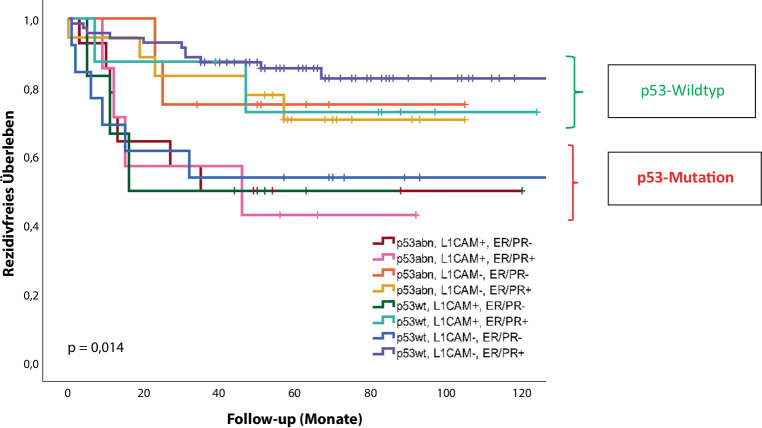

Die Studie bestätigt, dass p53 eine Schlüsselstellung einnimmt und der wichtigste Diskriminator zwischen guter/intermediärer Prognose (p53-negative Tumoren) und schlechtem Outcome (p53_mut_) ist. Dies ist eindrucksvoll Abb. 1 zu entnehmen. Die zusätzlichen Faktoren wie L1CAM und die guten alten Hormonrezeptoren modulieren lediglich marginal. Dabei beeinflussten L1CAM-Negativiät und Hormonrezeptorpositivität das klinische Outcome zum Besseren, L1CAM-Positivität und Hormonrezeptornegativität beeinflussten das Outcome ungünstig.

## Schlussfolgerung der Autoren

Der Einsatz von präoperativen IHC-Biomarkern hat neben der ESMO-ESGO-ESTRO-Risikoklassifizierung und neben dem Lymphknotenstatus eine wichtige prognostische Bedeutung. Für die tägliche klinische Praxis könnte die p53/L1CAM/ER/PR-Expression als Indikator für das chirurgische Staging und die Verfeinerung der selektiven adjuvanten Therapie durch Einordnung in die ESMO-ESGO-ESTRO-Risikoklassifizierung dienen [19].

## Kommentar

Die genomische Einteilung hat zu vier Subgruppen geführt, die eine unterschiedliche Prognose anzeigen und sich bezüglich des Ansprechens z. B. auf Immuntherapie deutlich unterscheiden. Faktoren, wie die bekannten Hormonrezeptoren oder das Adhäsionsmolekül L1CAM, haben einen zusätzlichen Einfluss, aber die wichtigste Unterscheidung bietet p53.

Auch heute kann hier schnell und preiswert schon in jedem Op.-Präparat die Unterscheidung getroffen werden. Die anderen Untersuchungen sind aufwendiger und teurer. An ihnen werden wir in Zukunft aber wohl nicht vorbeikommen, obwohl leider die deutsche Leitlinie aufgrund zeitraubender formaler Prozesse eine der wenigen Leitlinien ist, die hier noch kein Update publiziert haben. Hier sei auf die ESGO/ESMO-Leitlinie [17] verwiesen bzw. auf die NCCN-Leitlinie (Version 3/21; [19]). Die genomische Klassifizierung wird also in Zukunft eine Entscheidungsgrundlage bieten für den Ausschluss einer Hochrisikosituation (p53_wt_) bzw. das Vorliegen einer Hochrisikosituation (p53_mut_). Dies wird aller Voraussicht nach auch eine wichtige Entscheidungshilfe für die Indikation zur Lymphonodektomie sein, den Einsatz der postoperativen perkutanen statt der Brachytherapie und die Indikation zur Chemotherapie klären.

## Fazit

Bei Vorliegen von POLE-Mutationen handelt es sich wahrscheinlich um Patientinnen, die nicht von einer adjuvanten Therapie profitieren. MSI-Patientinnen haben eine intermediäre Prognose, sprechen aber auf Immuntherapien sehr gut an und die Subgruppe der NSMP-Patientinnen bedarf in Zukunft sicher noch einer verfeinerten Subgruppenanalyse. Klinische Studien werden in den nächsten Jahren zeigen, ob der Weg der genomischen Klassifikation bezüglich Prognose und Therapieentscheid der richtige ist.

*Simone Marnitz, Köln*
